# Carbon partitioning in sugarcane (*Saccharum* species)

**DOI:** 10.3389/fpls.2013.00201

**Published:** 2013-06-18

**Authors:** Jianping Wang, Spurthi Nayak, Karen Koch, Ray Ming

**Affiliations:** ^1^FAFU and UIUC SIB Joint Center for Genomics and Biotechnology, Fujian Agriculture and Forestry UniversityFuzhou, China; ^2^Agronomy Department, Genetics Institute, University of FloridaGainesville, FL, USA; ^3^Plant Molecular and Cellular Biology Program, University of FloridaGainesville, FL, USA; ^4^Horticultural Sciences Department, University of FloridaGainesville, FL, USA; ^5^Department of Plant Biology, University of Illinois at Urbana-ChampaignUrbana, IL, USA

**Keywords:** sugarcane, carbon partitioning, source-sink system, sucrose, cellulose, phloem, invertase, UDP-glucose

## Abstract

Focus has centered on C-partitioning in stems of sugarcane (*Saccharum* sp.) due to their high-sucrose accumulation features, relevance to other grasses, and rising economic value. Here we review how sugarcane balances between sucrose storage, respiration, and cell wall biosynthesis. The specific topics involve (1) accumulation of exceptionally high sucrose levels (up to over 500 mM), (2) a potential, turgor-sensitive system for partitioning sucrose between storage inside (cytosol and vacuole) and outside cells, (3) mechanisms to prevent back-flow of extracellular sucrose to xylem or phloem, (4) apparent roles of sucrose-P-synthase in fructose retrieval and sucrose re-synthesis, (5) enhanced importance of invertases, and (6) control of C-flux at key points in cell wall biosynthesis (UDP-glucose dehydrogenase) and respiration (ATP- and pyrophosphate-dependent phosphofructokinases). A combination of emerging technologies is rapidly enhancing our understanding of these points and our capacity to shift C-flux between sucrose, cell wall polymers, or other C-sinks.

Sugarcane (*Saccharum sp*.) is a large, perennial grass, mainly grown in tropical or subtropical regions for sugar and recently also for biofuel production. In the past 20 years, sugarcane has gained increased global prominence because of its superior potential for use as an alternative renewable energy source. Sugarcane belongs to the Poaceae family and is a member of Andropogoneae tribe, along with maize and sorghum. As a C4 plant, sugarcane is one of the most efficient crops in converting solar energy into chemical energy. Carbon (C) partitioning is a critical process in distributing the chemical energy converted by plant through photosynthesis. Generally, photosynthesis and C assimilation occur in chloroplasts of leaf mesophyll cells and additionally in bundle sheath cells in C4 plants. The C fixed during photosynthesis is converted into sugar or sugar derivatives in photosynthetic source cells then is distributed to distal sink cells. After long distance transportation in phloem, the sugars are imported in sink tissues and undertake two different fates: consumption and storage (Poorter and Villar, 1997). Typically about 35–40% of sugars (Hall and Rao, 1999) are consumed by the living cells to provide energy for cell growth, including cell expansion, division, differentiation, nutrient uptake, and maintenance during plant development. Some portion present in cells as metabolic intermediates such as simple sugars, amino acids, organic acids, etc. The remaining sugar can be stored as such in vacuoles or fixed in polymers that can either be remobilized (such as starch in plastids), or added to structural biomass (such as cellulose, hemicelluloses, and lignin).

## THE SOURCE-SINK SYSTEM IN SUGARCANE

Sugarcane, primarily used for sugar production, has a unique source-sink system. Its stem sinks store photosynthate as soluble disaccharide, sucrose, which can reach exceptionally high concentrations, up to 650 mM (Welbaum and Meinzer, 1990) or 18% of stem fresh weight in commercial sugarcane varieties (Inman-Bamber et al., 2011). In contrast, most other plant stems store C as insoluble polysaccharides such as starch or cellulose with a low concentration of sucrose. During sugarcane maturation, the fate of assimilated C shifts from that of insoluble and respiratory components, to sucrose, an osmotically active storage solute (Whittaker and Botha, 1997). Another distinctive feature of sugarcane is that sucrose storage occurs in the stalk (culm) parenchyma cells (Rae et al., 2005a, b; Uys et al., 2007) and not in terminal sink organs such as tubers, grains, or fleshy fruits. Furthermore, unlike many other systems, sugarcane accumulates sucrose both inside and outside the cells, in the symplast and apoplast, respectively (Welbaum and Meinzer, 1990).

**FIGURE 1:**
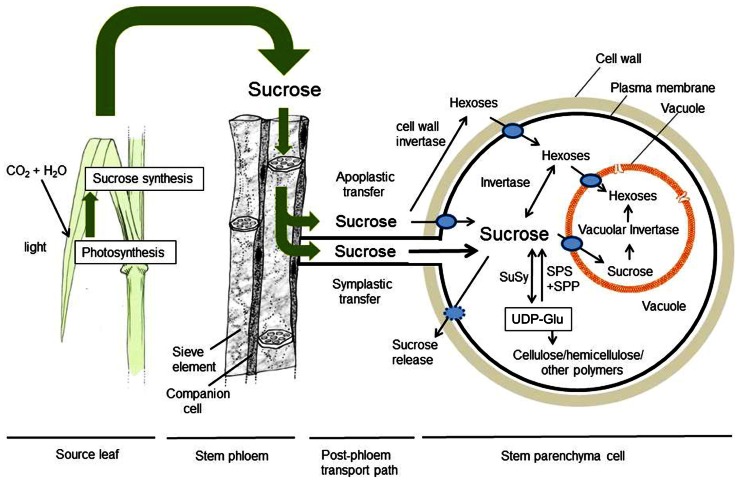
**Process diagram for sucrose movement and metabolism in sugarcane from its synthesis in source leaves to its deposition in stems.** Sucrose movement is shown with green arrows, and its subsequent metabolism and compartmentalization with narrow black arrows. Transporters are shown with blue ovals, cell wall is designated with a wide tan circle, and the vacuole is shown in orange. Sucrose synthesized in photosynthetic leaves is translocated in phloem to stem parenchyma cells, where its post-phloem transfer can follow two paths: symplastic (through plasmodesmata, as thought to predominate in the mature sugarcane internodes) and/or apoplastic (through the cell wall space considered a possible contributor earlier in sugarcane stem growth). Sucrose can move unaltered to storage parenchyma by either path, but apoplastic transfer could involve sucrose hydrolysis to hexoses by cell wall invertase. Both hexoses and sucrose then enter parenchyma cells via transporters. Hexoses can also form from sucrose inside cells by either neutral invertases in the cytoplasm, or vacuolar acid invertases. Sucrose is stored both in vacuoles and cell wall space, the balance between them including transporters and sucrose release to the apoplast. Internal sucrose supplies and partitioning to competing C-sinks also involves balance with UDP-Glu (UDP-glucose), a precursor for cell wall biosynthesis. Key reactions include the reversible SuSy (sucrose synthase), SPS (sucrose-P-synthase), and SPP (sucrose-P-phosphatase) reactions, all having central roles in sugarcane storage cells and operating as shown.

During development, sucrose synthesized in photosynthetic sugarcane leaves is translocated via phloem to stem internodes (**Figure 1**), including both immature stem internodes, the meristematic growth sink and mature internodes, the storage sink. In many plants, meristematic sinks are source-limited and storage sinks are sink-limited (Smith and Stitt, 2007). If true for sugarcane, the immature stalks would be constrained by the extent of available photosynthate, while the mature sugarcane stalks would be limited by their capacity to import sucrose from leaves. Sucrose accumulation in sinks depends on the size and activity of sinks, and this in turn can enhance photosynthate production by relieving feedback repression at metabolic and transcriptional levels (Koch, 1996; McCormick et al., 2008). During maturation of commercial sugarcane cultivars, the leave photosynthetic activity decreases significantly, as culm sucrose content increases (McCormick et al., 2008, 2009) probably indicating sink regulation of source capacity (Watt et al., 2005; McCormick et al., 2006), though nitrogen deficiency in mature sugarcane leaves sometimes also causes photosynthesis depression.

Sucrose can be rapidly sucrose breakdown for respiration and then re-synthesized in sinks, which allows for a dynamic balance between storage and respiration or other uses (Wendler et al., 1990). During the sucrose degradation and re-synthesis cycle, C is partitioned into other competing metabolic sinks including respiratory pathways, cycling through hexos pools, organic acids and amino acids, proteins and cell walls (Botha et al., 1996; Whittaker and Botha, 1997). Extent of each depends on developmental changes in capacities of different sinks, feedback from sinks on photosynthetic rates, and level of sucrose supplies from source leaves (Hofmeyr and Rohwer, 2011). Typically, immature sugarcane tissues partition considerable C into protein and fiber, whereas mature culms partition C mainly to sucrose storage (Bindon and Botha, 2002). At the whole-plant level, the rapid metabolism of sucrose in sink tissues allows for quick responses to shifts in sucrose supply and demand, as well as enhancing the constant sink strength, which can aid continuity in removal of photoassimilates from source leaves and help minimize sugar repression of photosynthesis (Krapp et al., 1993; Koch, 2004).

Diverse means have therefore been employed to increase the sink strength of sugarcane stems. One of these is transformation of a bacterial sucrose isomerase (SI) gene in sugarcane to convert sucrose to isomaltulose, a metabolite not native to higher plants. When the bacterial SI was targeted to the vacuole in transgenic sugarcane, about 50% of the total sugar in mature culms was recovered as isomaltulose (Wu and Birch, 2007). Since sucrose accumulation continued in SI transgenic sugarcane, the total sugars (sucrose + isomaltulose) nearly doubled in some instances. Further detailed characterization of the transgenic line cell cultures illustrated multiple changes consistent with greater sugar accumulation including reduced activities of extracellular invertase, symplastic sucrose-cleavage enzymes, increased sucrose biosynthesis, possibly coordinated through the trehalose-6-phosphate and sucrose-non-fermenting-1-related protein kinase 1 (Wu and Birch, 2010). This “sugarbooster” effect did not reduce partitioning to cell wall constituents (Wu and Birch, 2007, 2010). A similar effect was also achieved by over-expressing a fructosyl-transferase gene from the *Cynara scolymus* in transgenic sugarcane. Resulting plants converted 78% of culm sucrose to fructans, which led to a 63% greater total sugar content (Nell, 2007). These studies showed that additional metabolic sinks for sucrose could increase sink capacity, and lead to expected enhancement of photosynthesis and overall sugar accumulation (Koch, 1996, 2004).

## SUCROSE ACCUMULATION IN SUGARCANE

Sucrose can be synthesized in both photosynthetic and storage cells by sequential action of two enzymes: sucrose phosphate synthase (SPS) and sucrose phosphate phosphatase (SPP). The SPS reaction forms sucrose-P from fructose-6-P and UDP-glucose (UDP-Glu), and proceeds strongly in the synthetic direction due to rapid conversion of sucrose-P to sucrose by SPP (Botha and Black, 2000). In sugarcane, SPS activity correlates with sucrose content in diverse genotypes (Grof et al., 2007), though over-expression of SPS alone in transgenic sugarcane plants has not led to improved sucrose yields (Vickers et al., 2005).

Despite its name, the reversible reaction of sucrose synthase (SuSy) operates primarily in the degradative direction *in vivo*. Sucrose is cleaved into fructose and UDP-Glu, typically used for a combination of respiration and polymer (starch or cell wall constituents) biosynthesis (Lingle and Smith, 1991; Buczynski et al., 1993). SuSy is active in young internodes of sugarcane stems (Goldner et al., 1991; Schafer et al., 2004), and is negatively correlated with sucrose and positively correlated with hexose levels (Verma et al., 2011). Over-expression of a cotton SuSy gene in poplar enhanced C-partitioning to cellulose synthesis and altered the cell wall crystallinity (Coleman et al., 2009).

Sugarcane culms deposit sucrose in both the stem parenchyma cell vacuoles and the apoplast surrounding these cells (**Figure 1**). Parenchyma cells in mature sugarcane stalks can accumulate sucrose to levels having an osmotic potential of -2.2 MPa (Welbaum and Meinzer, 1990). Therefore, these cells have adapted to a potentially wide range of turgor, which may be under systemic regulation. The increased turgor in parenchyma cells may induce sugar release back into apoplast as part of a dynamic equilibrium between uptake and turgor-induced leakage (reviewed by Moore, 1995). One mechanism of turgor regulation could be to partition a fraction of cell solutes into the apoplastic space, allowing storage tissue to maintain a low gradient of solute concentrations between the apoplastic and symplastic compartments. This scenario could easily include the emerging roles of SWEET-type transporters for facilitated equilibration across membranes (Chen et al., 2012). Apoplastic back-flow of sucrose to the xylem and phloem would be minimized by the presence of a barrier to solute movement provided by the suberized, lignified sclerenchyma cells surrounding vascular bundles (Walsh et al., 2005). Xylem sap contains no detectable sucrose despite its traversing these storage tissues (Welbaum et al., 1992).

Sucrose is transferred from phloem to storage cells in the culm, probably through both the symplast and apoplast (Rae et al., 2005a, b; **Figure 1**) and predominantly the symplast in mature internodes (Patrick, 1997; Patrick et al., 2013). The subsequent compartmentation of sugars between the apoplast, cytosol, and vacuole is an important feature of storage in sugarcane stem’s parenchyma cells (Rae et al., 2009). Sucrose unloaded from phloem into the apoplast can follow two paths to vacuoles of parenchyma cells (**Figure 1**). In one path, sucrose is transported directly into parenchyma cells by sucrose transporters of the plasma membrane, then into the vacuole, mostly under low turgor conditions. In the other path, sucrose in the apoplast is hydrolyzed by apoplastic acid invertase into glucose and fructose. These in turn are transported by hexose carriers, with sucrose re-synthesis occurring in the cytoplasm prior to vacuolar storage (**Figure 1**).

Transporters of the plasma membrane appear to play an important role not only in phloem loading and unloading, but also in transferring sucrose between apoplastic and symplastic compartments (Riesmeier et al., 1994; Burkle et al., 1998; Braun and Slewinski, 2009; Chen et al., 2012). A survey of thousands of transcript sequences from maturing sugarcane culms revealed that transcripts for sugar metabolizing enzymes are relatively rare in maturing culms while transcripts for sugar transporters are very abundant (Carson et al., 2002; Casu et al., 2003). Also, mRNAs for a *ShSUT1* transporter gene of a sugarcane hybrid were abundant in both source leaves and sink stems actively accumulating sucrose (Rae et al., 2005a, b). The presence of these *ShSUT1* transcripts at the periphery of the vascular parenchyma and mestome sheath cells, instead of in the phloem itself, is consistent with a role other than that of direct phloem loading. Instead, their function may contribute to a biochemical barrier that inhibits sucrose apoplastic back-flow out of tissues and also aid retrieval of sucrose released to the apoplast (Rae et al., 2005a, b, 2009). The *ShSUT1* gene product may thus have a role in the partitioning of sucrose between vascular tissue and storage sites in sugarcane stem parenchyma cells (Reinders et al., 2006).

In addition to the genes noted above for sucrose synthesis and transport, those encoding invertases have been suggested as key regulators for sucrose accumulation in sugarcane stem. There are three types of invertases: neutral invertases in the cytoplasm, insoluble acid invertases in the cell wall space, and soluble acid invertases in the vacuole. Soluble acid invertase activities are usually high in rapidly growing tissues, such as root apices and immature stem internodes. In sugarcane, soluble acid invertase is most active in immature internodes that accumulate the least sucrose, and minimally active in maturing internodes with high sucrose content. Although suppression of the soluble acid invertase also increased sucrose content in sugarcane suspension cell culture (Ma et al., 2000), a similar response was not evident for the overall sucrose content of mature, transgenic sugarcane plants (Botha et al., 2001). The balance between soluble acid invertase and SPS activities influences the sucrose accumulation in sugarcane internodes, favoring sucrose storage when SPS predominates given soluble acid invertase at below critical threshed concentration (Zhu et al., 1997).

Effects of down-regulating neutral invertase activity reduced by 40% were also tested in transgenic lines (Rossouw et al., 2010). Both sucrose and hexoses content rose. Specifically, sucrose content increased by 25 and 14% in the immature and mature culms, respectively, but this benefit was outweighed by a severe reduction in plant vigor (Rossouw et al., 2010). The reduced neutral invertase in these stems appeared to be compensated by an increase in SuSy activity (Rossouw et al., 2010).

Cell wall invertase is often considered a gateway for the entry of sucrose into the cells of juvenile tissues that have an apoplastic path of phloem loading (reviewed by Moore, 1995). Increases in cell wall invertase activity are associated with higher sucrose content in sugarcane (Lingle, 1989). Greater cell wall invertase activity in high-sugar genotypes may operate by enhancing sucrose unloading into the internode tissue (Chandra et al., 2012).

## CARBON PARTITIONING TO CELL WALL SYNTHESIS

Though sucrose content in the sugarcane culm ranges from 14 to 42% of the culm dry weight (Whittaker and Botha, 1997), the majority of carbohydrate in sugarcane is lignocellulose, a major component in the cell wall. The latter may ultimately be a more effective for C reservoir, since they rarely re-enter active metabolism, and have less osmotic effect on cells than would sucrose. As cell elongation and sucrose accumulation ceases in the maturing sugarcane internodes, there is a major increase in cell wall thickening and lignification (Botha and Black, 2000). The predominant polysaccharide component in culm cell walls is cellulose (Lingle et al., 2008; Sainz, 2009). Cellulose accounts for 28–30% of the above-ground dry matter in typical forage grasses (Theander and Westerlund, 1993), 42–45% in wood (Smook, 1992), and about 42–43% in sugarcane and energy cane cultivars (Kim and Day, 2011). As the most abundant reservoir of C in nature, cellulose and other polymers can be prominent competing sinks for C in sugarcane.

Cellulose is a strong, essentially irreversible C-sink in plants (Manners, 2011). Cellulose synthesis is catalyzed by enzyme complexes of cellulose synthase (CesA), arranged in rosette formations at the bases of growing cellulose fibrils. The CesA reactions are coordinated with those of SuSy that operates in the degradative direction in supplying UDP-Glu substrates at cellulose synthesis sites. CesA mutants of *Arabidopsis* that result in disassembled CesA complexes show reduced cellulose synthesis capacity, defective elongation growth, and collapsed xylem elements (Arioli et al., 1998). Balanced C-partitioning to cell wall biosynthesis is thus essential. Elevated SPS activity is consistently correlated with high rates of cellulose synthesis and secondary wall deposition, since SPS would allow re-synthesis of sucrose substrate for cell wall biosynthesis and retrieval of any excess fructose produced during the path to cellulose formation (Babb and Haigler, 2001). A decreased cellulose synthetic capacity is often partly compensated by increases in production of cell wall pectin and hemicellulose (Sato et al., 2001). During culm maturation in sugarcane, the cellulose synthesis is regulated through coordinated expression of diverse genes and gene families including those encoding cellulose synthases, cellulose synthase-likes, enzymes for lignin biosynthesis, and a range of other genes identified in clusters from expression profiles of different tissues (Casu et al., 2007).

Potential for altering C-partitioned to respiration in sugarcane has also been explored by transgenic manipulation of a regulatory point in glycolysis. Two enzymes in plant: ATP-dependent phosphofructokinases (PFK) and pyrophosphate-dependent pyrophosphate: fructose 6-phosphate 1-phosphotransferase (PFP), operate glycolysis at such a point, both forming fructose 1,6-bisphosphate. Modification of either enzyme could potentially divert hexose phosphates to respiratory pathways. Whole transgenic sugarcane stalks with reduced PFP had enhanced sucrose and fiber content when young (Groenewald and Botha, 2008). The partitioning to sucrose was no longer evident at maturity, presumably because metabolite equilibration had followed age-related decreases in glycolytic rates. However, results did demonstrate that PFP activity may constrain sucrose accumulation in immature internodes by regulating ratios of hexose phosphates to triose phosphates, and thus support a critical role for this enzyme in glycolytic C flow (van der Merwe et al., 2010). A kinetic model investigating the effect of enzyme level changes on sucrose partitioning inferred that sucrose synthesis and storage is mostly depended on one of SuSy isozymes and the SuSy isozymes are negatively controlling both PFK and PFP over sucrose storage flux (Uys et al., 2007). Other avenues for modulating this process include the ATP-dependent PFK and pyruvate kinase reactions, but both steps are tightly regulated. Effective manipulations to increase metabolic flux will need to include both the removal of feedback control and increased demand for C.

UDP-Glu, a nucleotide sugar central to diverse pathways of polysaccharide biosynthesis, leading to starch, cellulose, and others is another point of potential manipulation. About 10 major monosaccharides in cell wall polymers are converted from glucose through UDP-Glu related interconversion pathways. UDP-Glu is thus the precursor for most cell wall polysaccharides such as cellulose, hemicellulose, and callose (Gibeaut, 2000; Kleczkowski et al., 2004; Joshi and Mansfield, 2007). UDP-Glu is also the substrate for sucrose synthesis by SPS. To reduce use of UDP-Glu for cell wall biosynthesis, transgenic sugarcane was used to down-regulate UDP-Glu dehydrogenase activity, which catalyzes conversion of UDP-Glu to UDP-glucuronate, a precursor for both hemicelluloses and pectin formation (Bekker, 2007). Increases in both sucrose accumulation and SPS activity suggested an altered C-flux toward sucrose. Anticipated decreases in cell wall components were not observed, possibly due to partial compensation by activation of the myoinositol oxygenation pathway for cell wall precursor synthesis (Bekker, 2007). The mechanisms regulating cell wall biosynthesis and source-sink relations in sugarcane will be crucial constituents of any efforts to alter C-partitioning between fiber and sugar in the culm.

## CONCLUDING REMARKS

Sucrose synthesis in source tissue, its translocation, and its partitioning between storage, respiration, and biosynthesis are systemically coordinated in plants (Ayre, 2011). Although considerable effort has been made to understand C-partitioning in sugarcane, the biochemical process is far from fully understood. Challenges have included its complex genome, high polyploidy level, and limited genome resources, all of which have hindered forward genetics studies. With the advance of high throughput DNA sequencing, development of gene-expression technologies, and enrichment of genetic/genomics resources for *Saccharum*, the regulatory networks of C-partitioning in different sinks of sugarcane can be elucidated systematically. Translational genomics and other comparative tools will also allow advances in other systems to be used for developing testable hypotheses in sugarcane and vice versa. The hybrids between high sucrose sugarcane cultivar and *S. spontaneum* with low sucrose content and high fiber content have a range of sugar to fiber ratios and this genetic variation could also provide insights into how carbon is partitioned between sucrose and fiber. Future research can be aimed at understanding the molecular and physiological processes underlying accumulation of sucrose and lignocellulosic biomass, as well as manipulation of the balance between them.

## Conflict of Interest Statement

The authors declare that the research was conducted in the absence of any commercial or financial relationships that could be construed as a potential conflict of interest.
